# Adaptation and psychometric validation of the Turkish version of the Grief Support in Healthcare Scale: a methodological study

**DOI:** 10.1136/bmjopen-2025-116014

**Published:** 2026-07-03

**Authors:** Buket Şimşek Arslan, Remziye Semerci Şahin

**Affiliations:** 1 Nursing Department, Faculty of Health Sciences, Burdur Mehmet Akif Ersoy Universitesi, Burdur, Turkey; 2 School of Nursing, Pediatric Nursing, Koç University, Istanbul, Turkey

**Keywords:** Death, Hospice and Palliative Care Nursing, Decision Making

## Abstract

**Abstract:**

**Objectives:**

The study aimed to adapt the Grief Support in Healthcare Scale (GSHCS) for use in the Turkish context and evaluate its psychometric properties among nurses.

**Design:**

This study is a methodological study.

**Setting:**

This study was conducted in Turkey.

**Participants:**

The pilot study included 30 nurses for preliminary testing and test-retest reliability. The main study comprised a convenience sample of 355 nurses.

**Outcome measures:**

Content validity was evaluated by a panel of experts using the Content Validity Index (CVIs). Construct validity was assessed through exploratory factor analysis (EFA) and confirmatory factor analysis (CFA). Internal consistency was examined using Cronbach’s α and split-half reliability, while convergent validity was tested via correlations with the Professional Bereavement Scale (PBS).

**Results:**

CVIs were strong (Item-Level Content Validity Index=0.99; Scale-Level Content Validity Index=0.94). EFA identified a three-factor structure explaining 58.33% of the total variance. CFA confirmed the three-factor model (χ^2^(83) = 338.204, χ^2^/df=4.075, root mean square error of approximation=0.093, Goodness of Fit Index=0.892, Comparative Fit Index=0.922, Tucker-Lewis Index=0.901, Incremental Fit Index=0.923, Normed Fit Index=0.900). Cronbach’s α was 0.888 for the total scale. All subdimensions showed significant positive correlations with PBS-Short-term Bereavement Reactions Subscale (r=0.176–0.401).

**Conclusion:**

The Turkish version of the GSHCS is a valid and reliable tool for assessing nurses’ perceptions of grief support in healthcare settings. The scale retains its conceptual structure and demonstrates sound psychometric properties, making it suitable for clinical research and organisational assessments in Turkey.

**STRENGTHS AND LIMITATIONS OF THIS STUDY**

- This study followed a rigorous methodological approach for the cultural adaptation and psychometric validation of the Grief Support in Healthcare Scale within the Turkish context.
- Content validity was ensured through expert panel evaluation, and construct validity was robustly tested using both exploratory and confirmatory factor analyses, strengthening the methodological rigour.
- The scale demonstrated strong internal consistency and acceptable convergent validity, supporting its reliability for assessing nurses’ perceptions of grief support in healthcare settings.
- The sample was recruited through convenience sampling via online platforms, which may limit the representativeness and generalisability of the findings.
- Convergent validity was assessed using a single external instrument, limiting triangulation of the construct.

## Introduction

 Patient death is an inevitable and recurrent experience in nursing practice, with profound implications for nurses’ professional functioning and psychological well-being. Understanding how grief manifests in healthcare settings and how institutional and collegial support can mitigate its adverse effects is therefore of critical importance for nursing research and practice.

### Nurses’ experiences of grief and the need for support

Nurses working in diverse healthcare settings, including emergency departments, intensive care units, palliative care services, nursing homes and oncology clinics, are frequently confronted with the death of their patients. Such encounters inevitably elicit grief reactions, which may be acute or cumulative.^1 2^ Repeated exposures to patient deaths have been associated with chronic grief responses that can accumulate over time and negatively affect both professional functioning and personal well-being.^3 4^ These unresolved grief reactions may contribute to compassion fatigue,^4 5^ diminished capacities for therapeutic engagement,^4 6^ professional burnout^7 8^ and lower levels of job satisfaction.^8 9^

From a sociocultural perspective, grief is not only an individual experience but also a socially regulated process. Societal norms determine which losses are deemed legitimate, who is considered entitled to grieve, the acceptable forms of expressing grief and which bereaved individuals receive communal support. These socially constructed rules create the phenomenon of disenfranchised grief, where individuals’ grief reactions are invalidated or unrecognised.^10^ Within the nursing profession, disenfranchised grief is particularly salient because patient deaths are often perceived as ‘part of the job’, leading to the minimisation or neglect of nurses’ emotional needs. Recognising the relational bond that nurses develop with patients is therefore essential to understanding the depth and complexity of their grief responses. Furthermore, active acknowledgement of loss and the inclusion of nurses in grief rituals represent critical avenues for legitimising their grief and extending formal and informal support mechanisms.^11^

Given the significant emotional burden of patient deaths, both individual coping strategies and institutional responsibilities are required to mitigate the adverse consequences of grief among nurses. Institutions play a vital role in providing structured support systems, such as professional counselling, peer debriefing sessions, training in communication about end-of-life care, education in grief theory and opportunities to participate in memorial activities.^4 12^ Research consistently demonstrates that effective workplace support reduces burnout, alleviates grief-related distress and enhances resilience among nurses.^13^,^15^ In addition, colleagues are recognised as a critical source of informal support, providing spaces for shared reflection, companionship and therapeutic communication grounded in common experiences.^16 17^ These findings underscore the centrality of workplace support systems in sustaining nurses’ well-being, professional motivation and quality of patient care.^2^

### Need for a measurement tool in Turkey and aim of the study

In Turkey, several psychometric instruments have been developed or adapted to evaluate grief-related processes. These include scales assessing grief reactions,^18^ prolonged grief disorder,^19 20^ grief-related functional impairment,^21^ relational aspects of grief^22^ and meaning reconstruction after loss.^23^ While these tools provide valuable insights into various dimensions of grief, none specifically focuses on grief support within healthcare contexts. This gap is particularly critical for the nursing profession, where grief is both pervasive and often disenfranchised. Considering the emotional burden that nurses experience in response to patient deaths and the complex relational dynamics inherent in healthcare environments, there is a pressing need for a culturally adapted, psychometrically sound instrument capable of evaluating grief support in healthcare. Such a tool would facilitate the identification of support needs, enable targeted interventions and contribute to the formulation of institutional policies to improve nurses’ psychosocial well-being. The Grief Support in Healthcare Scale (GSHCS) was developed by Anderson, Ewen and Miles (2010) specifically to measure healthcare workers’ perceptions of the grief support they receive in workplace settings. The scale was originally developed and validated among certified nursing assistants working in long-term care facilities in the USA, a context in which repeated patient loss is a normative occupational experience. The development process followed rigorous psychometric procedures, including item generation grounded in the disenfranchised grief framework, expert review and confirmatory factor analysis (CFA), resulting in a 15-item, three-factor instrument: recognition of the relationship, acknowledgement of the loss and inclusion of the griever.^11^ A key strength of the GSHCS that distinguishes it from existing grief-related instruments is its explicit focus on perceived institutional and collegial support rather than on individual grief reactions or symptomatology. While existing scales assess grief responses,^18^,^23^ the GSHCS uniquely captures the relational and organisational dimensions of grief support in professional healthcare environments, making it particularly suited for identifying systemic gaps and informing workplace interventions. To date, a cross-cultural adaptation of the GSHCS has been conducted in South Korea, where the scale was validated among frontline nurses during the COVID-19 pandemic.^24^ Notably, the Korean validation study omitted the ‘Inclusion of the Griever’ subscale owing to cultural norms surrounding funeral attendance, highlighting the importance of culturally sensitive adaptation in diverse healthcare contexts.^24^ Despite this growing international interest, no validated version of the GSHCS exists for Turkish-speaking populations, representing a critical gap given the distinct sociocultural and institutional context of grief support in Turkey. Accordingly, the present study aims to adapt the GSHCS, originally developed by Anderson *et al*^11^ into Turkish and to examine its psychometric properties among nurses working in diverse clinical settings in Turkey.

## Method

### Study design

This methodological and cross-sectional study aimed to assess the measurement properties of the Turkish version of the GSHCS among nurses. The study was designed and reported in accordance with the COnsensus-based Standards for the Selection of health Measurement INstruments guidelines for the evaluation of patient-reported outcome measurement instruments.^25^

### Study sample

There are different suggestions for calculating sample size for scales. Tabachnick *et al*^26^ suggest that the participant/item ratio should be 10:1, while Schreiber *et al*^27^ suggest this ratio to be 20:1. According to another view, in scale validity and reliability studies, the determination of sample size is based on the 100 rule: <100: very low; 100–200: low; 300: good and 500–1000: very good. The general view is that, for effective factor analysis, researchers should include at least 20 participants per item.^28 29^ To conduct the validity and reliability study of the GSHCS (15 items), when 20 nurses were taken per item, the sample size for the study was calculated as at least 300 nurses. A total of 355 nurses participated in the study.

### Patient and public involvement

No patients or members of the public were involved in the design, conduct, reporting or dissemination of this research. This study focused on the psychometric validation of a scale among registered nurses, who participated solely as survey respondents and were not involved in setting the research agenda or outcome measures.

### Study setting

This study was conducted online between March and April 2025. A convenience sampling strategy was employed; the study sample consisted of nurses working in healthcare settings who volunteered to participate. The survey link was distributed through multiple online channels, including WhatsApp groups, LinkedIn, Facebook groups, Instagram and the email lists of nursing professional associations. Nurses who were accessible via these platforms and agreed to participate completed the online questionnaire voluntarily.

#### Inclusion criteria

Nurses were included if they worked in healthcare settings, had at least 1 year of professional experience, had experienced at least one patient death during their career and were accessible via the specified online platforms.

#### Exclusion criteria

Nurses with <12 months of professional experience, those who filled in incomplete data and those who wanted to leave the study at any stage were excluded from the study.

### Data collection tools

The Nurse Information Form, the GSHCS and the Professional Bereavement Scale (PBS) were used to collect data.

#### Nurse information form

The form was developed by the researchers based on a review of the relevant literature on grief in nursing and workplace bereavement support.^2 11 12^ It consists of seven items evaluating nurses’ sociodemographic characteristics, grief education status and the availability of grief support services in their workplace.

#### Grief support in healthcare scale

The GSHCS was developed by Anderson *et al* (2010) to assess grief support in healthcare. The scale consists of 15 items and is scored on a five-point Likert scale (strongly disagree=1 point; disagree=2 points; undecided=3 points; agree=4 points and strongly agree=5 points). GSHCS is a three-factor scale: Component 1, recognition of the relationship (Items 1, 2, 3, 4 and 5); Component 2, acknowledgement of the loss (Items 6, 7, 8, 9 and 10); Component 3, inclusion of the griever (Items 11, 12, 13, 14 and 15). The scale score is determined by calculating the mean scores. A higher score indicates a higher level of grief support. Cronbach’s α values for the scale’s internal consistency reliability were 0.89, 0.86 and 0.78, respectively.^11^ Permission was obtained from Anderson *et al* to conduct the validity and reliability analyses of the scale.

#### The professional bereavement scale

The PBS was developed by Chen and Chow to assess the grief experiences of healthcare professionals.^30^ The Cronbach’s α value for the Short-term Bereavement Reactions (PBS-SBR) Subscale was found to be 0.96 and the Cronbach’s α value for the Accumulated Global Changes (PBS-AGC) Subscale was found to be 0.94. The Cronbach’s α values for the PBS-SBR and PBS-AGC subscales of the scale adapted to the Turkish language and culture are 0.86 and 0.89, respectively. PBS-SBR measures the acute reactions of healthcare professionals to loss. It is a five-point Likert-type scale with 17 items. Each item in the PBS-SBR subscale is scored from 0 to 4, with ‘0’ indicating that the described behaviour is experienced at a minor level and ‘4’ indicating that it is experienced as extremely severe.

The PBS-AGC subscale measures the long-term effects of patient loss on healthcare professionals. This scale is also a five-point Likert-type scale consisting of 15 items. Each item in the PBS-AGC subscale is scored from 0 to 4, with ‘0’ indicating that the described behaviour is not experienced and ‘4’ indicating that it is experienced very much. There is no cut-off point for either subscale. As the score obtained from the subscale increases, it is interpreted that the participant’s experiences of accumulated global changes described increase.^31^ Permission to use the Turkish version of the PBS was obtained from Eryüksel and Özbaş, the developers of the Turkish adaptation, prior to data collection.^31^

### Data collection

Data were collected online between March and April 2025. Participants were reached through a multi-channel recruitment strategy targeting nursing professionals. Prior to distribution, permission was obtained from the administrators of all relevant groups and platforms. The survey link was shared via WhatsApp groups and Facebook groups exclusively composed of nursing professionals, as well as through LinkedIn and Instagram. Representatives of nursing professional associations also disseminated the survey link through their dedicated WhatsApp groups for healthcare workers. Nurses who encountered the survey link through any of these channels and met the inclusion criteria were directed to the online data collection form hosted on Google Forms. The first page of the form included a detailed informed consent statement outlining the study’s purpose, the voluntary nature of participation and the right to withdraw at any time without justification. Only participants who provided informed consent could proceed to the survey items. To ensure data integrity, the Google Forms setting was configured to allow only one submission per email address, preventing duplicate responses. Response timestamps were also reviewed to identify and exclude inattentive or incomplete submissions. All nurses who accessed and consented to the survey completed it in full, resulting in a 100% completion rate.

### Translation process

The translation process followed the WHO’s guidelines.^32^ The steps involved were translation, back-translation, review by a panel of experts and implementation of a pilot study. Two linguists with proficiency in Turkish and English and unfamiliar with the scale assessed the instrument’s language validity. The Turkish translations were evaluated and revised by the researchers and an external expert. The revised scale was further examined by a Turkish linguist to confirm conceptual and linguistic equivalence. The Turkish version was back-translated into English by two different linguists. This translation process ensured linguistic and conceptual agreement of the scale for the aim of this study.

### Content validity

Content validity, which is important in scale development, reflects how well a scale contains a set of appropriate items related to the concept being assessed. The WHO recommends assessing content validity in scale adaptation studies. Content Validity Index (CVI), which has two types as Item-Level Content Validity Index (I-CVI) and Scale-Level Content Validity Index (S-CVI), is the most frequently used measure in quantitative assessments.^33^ In accordance with the scale study guidelines, it is recommended to consult at least 3–20 experts to evaluate both I-CVI and S-CVI.^33 34^ Opinions of 10 experts were obtained in this study. Following the back translation, I-CVI and S-CVI were examined with expert evaluations as suggested by Polit and Beck.^34^ Experts were asked to rate the items on a scale from 1 (not relevant) to 4 (extremely relevant). From expert opinions, I-CVI and S-CVI values were calculated.

Experts were identified according to their psychiatric nursing, internal medicine nursing or paediatric nursing. Expert opinions were obtained from 10 experts with at least a PhD. All the experts are familiar with the concept of grief support. These experts also had experience in scale validity and reliability studies. CVIs were calculated for each item. I-CVI of all items was found to be at the desired level (>0.75). Also, the calculated S-CVI was 0.94. These results indicate unanimous agreement among experts. Revisions were made to the draft following expert recommendations. At this stage, no item was removed from the scale. Based on expert feedback, minor revisions were made to improve the clarity, comprehensibility and cultural appropriateness of several items. Some wording and sentence structures were refined to enhance readability and conceptual consistency. At this stage, no item was removed from the scale

The study was conducted with nurses working in healthcare settings in Turkey. There is no official grief support in the Turkish healthcare system for nurses. Furthermore, there is no regulation in Turkey on whether nurses can attend patient funerals. The ‘Inclusion of the Griever’ subscale was removed from the Korean version of the scale because healthcare workers rarely attend their patients’ funerals.^24^ Since there is no legal regulation regarding nurses’ participation in patient funeral ceremonies in Turkey, all items were included in the analysis to preserve the integrity of the scale. However, the cultural appropriateness of this subscale should be re-evaluated in future studies.

### Pilot test

The pilot study served two sequential purposes. The primary aim was item analysis: 30 nurses completed the scale and provided feedback on the readability, comprehensibility and clarity of each item. Items were reviewed based on participant responses and expert input, and the scale was finalised before the main study. As a secondary aim, following confirmation of content validity, test-retest reliability was assessed in this pilot subsample to evaluate the instrument’s temporal stability. The scale was administered two times, at day 0 and day 14, and the correlation between the two administrations was examined. Conducting this stability assessment at the end stage of the pilot phase, after item refinement, is consistent with recommended psychometric practice. The pilot test data were excluded from the main analysis.

### Data analysis

Data analysis was carried out using IBM SPSS Statistics V.28.0 (IBM, Corp, Armonk, NY, USA) and AMOS V.24.0 (IBM Corp., Armonk, NY, USA). Descriptive statistics, such as means and SD, were computed to summarise participants’ sociodemographic data and to report the total and subscale scores of the scale. Validity assessments included both content and construct validity. Content validity was determined using I-CVI and S-CVI according to the Davis method. The 355 participants were randomly split into two equal halves: exploratory factor analysis (EFA) was performed in the first half (n=178), and CFA was performed on the second half (n=177). For construct validity, the data were split into two parts: an EFA was performed on the first portion, and a CFA was carried out on the second. EFA used Principal Axis Factoring (PAF) with varimax rotation to identify the scale’s factor structure. The suitability of the data for factor analysis was evaluated using the Kaiser-Meyer-Olkin (KMO) measure and Bartlett’s Test of Sphericity. Prior to CFA, the distributional properties of the CFA subsample (n=177) were examined to select an appropriate parameter estimation method. Univariate normality was assessed by inspecting skewness and kurtosis for each item; values within ±2 for skewness and ±7 for kurtosis were considered indicative of acceptable univariate normality. Multivariate normality was evaluated using Mardia’s normalised multivariate kurtosis coefficient; values below 5.0 were considered consistent with the assumption of multivariate normality. Outliers were identified using Mahalanobis distance (D^2^) values computed for each case; observations with p_1_<0.001 were flagged as potential multivariate outliers and inspected for their influence on model fit prior to final analysis. Given that univariate normality was largely satisfied across items, maximum likelihood (ML) estimation was employed as the parameter estimation method, as ML is considered robust and produces efficient parameter estimates under approximate normality conditions. The hypothesised three-factor structure derived from EFA was tested as the baseline model. Item-level fit was evaluated through standardised factor loadings (λ≥0.50 considered acceptable) and the statistical significance of each loading (p<0.05). Model modification was guided by modification indices (MI); error covariance terms between theoretically related items within the same factor were freed sequentially when MI values exceeded 10.0, and the modification was conceptually justifiable. Model fit was evaluated using multiple indices: χ^2^, χ^2^/df ratio (acceptable≤5.0), root mean square error of approximation (RMSEA), standardised root mean square residual (SRMR) (acceptable<0.08; marginal<0.10), Goodness of Fit Index (GFI), Comparative Fit Index (CFI), Incremental Fit Index (IFI), Tucker-Lewis Index (TLI) and Normed Fit Index (NFI) (all acceptable≥0.90). Before conducting CFA, multicollinearity was examined using the variance inflation factor and tolerance values. Model fit was assessed with several goodness-of-fit indices, including χ^2^, df, χ^2^/df ratio, RMSEA, GFI, CFI, IFI, NFI and TLI. Reliability of the scale and its subscales was tested using Cronbach’s α. Composite reliability (CR) and average variance extracted (AVE) were calculated to assess construct reliability and convergent validity, respectively. CR values ≥0.70 and AVE values ≥0.50 were considered indicative of adequate reliability and convergent validity. Discriminant validity was evaluated using the Fornell-Larcker criterion, whereby the square root of each factor’s AVE was compared against the inter-factor correlations; AVE exceeding all corresponding inter-factor correlations was taken as evidence of discriminant validity. Additionally, split-half reliability was calculated to further confirm internal consistency. Hotelling’s T^2^ test was used to assess response bias. Pearson correlation analysis was used to evaluate convergent validity and to examine relationships among variables. All analyses were performed at the 95% confidence level, and results with p values <0.05 were considered significant.

## Results

### Sample characteristics

A total of 355 nurses participated in the study (table 1). The participants’ mean age was 31.30±8.12. Regarding gender, the sample was predominantly women, with 86.5% (n=307) identifying as women, and a majority held a bachelor’s degree (74.1%, n=263). For professional experience, 31.8% (n=113) of nurses had worked for 1–2 years, 23.4% (n=83) for 3–5 years, 12.4% (n=44) for 6–10 years and 32.4% (n=115) for more than 10 years. Participants were employed across a range of clinical settings, most commonly in inpatient services (30.1%, n=107), intensive care units (24.5%, n=87) and emergency departments (16.1%, n=57). When asked whether grief support services were provided at their institutions, only 20.0% (n=71) reported that such support was available and 28.5% (n=101) of nurses indicated that they had personally received grief support.

**Table 1:** The characteristics of the participant (n=355)

Variable	M±SD	
Age	31.30±8.12	
Gender	n	%
Female	307	86.5
Male	48	13.5
Education		
High school	13	3.7
Associate degree	8	2.3
Bachelor	263	74.1
Master	62	17.5
Doctorate	9	2.5
Professional experience		
1–2 years	113	31.8
3–5 years	83	23.4
6–10 years	44	12.4
>10 years	115	32.4
Type of the clinical settings		
Emergency	57	16.1
Intensive care unit	87	24.5
Palliative care	9	2.5
Inpatient	107	30.1
Outpatient	12	4.2
Others	80	22.5
Having grief support service in the hospital		
Yes	71	20.0
No	284	80.0

### Results of validity analysis

#### Content validity

Prior to the pilot implementation of the scale, a content analysis of the items and the overall scale was conducted based on feedback from 10 experts. The I-CVI was 0.99, reflecting a strong consensus among the experts. Additionally, S-CVI) was calculated as 0.94.

#### Test-retest reliability

Test-retest reliability was evaluated in the pilot subsample (n=30) with a 14-day interval between administrations. The Pearson correlation coefficient between the two time points was 0.857 (p<0.001), and the intraclass correlation coefficient was 0.937, indicating excellent stability of the scale.

### EFA results

An EFA was conducted to examine the underlying factor structure of the 15-item scale related to grief recognition and support among nurses. The KMO measure verified the sampling adequacy for the analysis, yielding a value of 0.862, which is considered ‘meritorious’. Bartlett’s test of sphericity was statistically significant (χ^2^=3132.532, p<0.001).

PAF with varimax rotation yielded a three-factor solution, accounting for 58.33% of the total variance. The first factor, labelled ‘Recognition of the Relationship’, accounted for 38.26% of the variance (eigenvalue=6.104) and included items reflecting how others understand the closeness of the nurses’ resident relationship (eg, ‘My co-workers understand how close I am to the residents’, loading=0.805). The second factor, ‘Acknowledgement of the Loss’, explained 12.88% of the variance (eigenvalue=2.421) and comprised items reflecting acknowledgement from social and professional contacts regarding the nurses’ grief following a resident’s death (eg, ‘My co-workers know that I have grief when residents die’, loading=0.921). The third factor, ‘Inclusion of the Griever’, accounted for 7.16% of the variance (eigenvalue=1.401), including items related to institutional practices such as holding memorial services and informing staff about resident deaths (eg, ‘My facility keeps me informed about the deaths of residents’, loading=0.661). All factor loadings exceeded 0.50, with the exception of Item 9 (loading=0.506) (table 2).

**Table 2:** Results of the exploratory factor analysis (n=355)

		Recognition of the relationship	Acknowledgement of the loss	Inclusion of the griever
Item 1	My family understands how close I am to the residents.	0.757		
Item 2	My friends understand how close I am to the residents.	0.814		
Item 3	My co-workers understand how close I am to the residents	0.805		
Item 4	My supervisors understand how close I am to the residents.	0.723		
Item 5	Family members of the residents understand how close I am to the residents	0.850		
Item 6	My family knows that I have grief when residents die.		0.904	
Item 7	My friends know that I have grief when residents die.		0.886	
Item 8	My co-workers know that I have grief when residents die.		0.921	
Item 9	My supervisors know that I have grief when residents die		0.506	
Item 10	Family members of the residents know that I have grief when residents die.		0.657	
Item 11	My facility often holds memorial services for residents who have died.			0.738
Item 12	I am often able to attend memorial services inside my facility.			0.771
Item 13	I am often invited to attend memorial services outside of the facility			0.797
Item 14	I am often able to attend memorial services for residents outside of the facility			0.720
Item 15	My facility keeps me informed about the deaths of residents			0.661
Eigenvalues		6.104	2.421	1.401
Explained variance (%)		38.26%	12.88%	7.16%

Total explained variance=58.33%, KMO coefficient=0.862, Bartlett‘s test=3132.532, p<0.001.

KMO, Kaiser-Meyer-Olkin.

### Reliability analysis results

The total scale has a Cronbach’s α of 0.888, exceeding the generally accepted threshold of 0.70 for reliability in psychometric instruments. Among the three subdimensions, the ‘Acknowledgement of the Loss’ subscale showed the highest reliability, with Cronbach’s α of 0.903. Item-total score correlations within this subscale ranged from 0.700 to 0.770, indicating strong internal consistency and homogeneous item content. The ‘Recognition of the Relationship’ subscale also demonstrated high reliability (α=0.874), with item-total correlations ranging from 0.595 to 0.694, further confirming the consistency of items assessing how others perceive the emotional closeness between nurses and residents. The ‘Inclusion of the Griever’ subscale yielded a Cronbach’s α of 0.796, reflecting acceptable reliability. Item-total correlations for this factor ranged from 0.429 to 0.563 (table 3).

**Table 3:** Results of the reliability analyses of the scale and correlations of the item total score (n=355)

Items	Cronbach α	Mean±SD	Item-total score correlation
1	Recognition of the relationship=0.874	3.40±1.03	0.694
2		3.50±1.01	0.688
3		3.66±1.00	0.645
4		2.98±1.07	0.622
5		3.43±1.04	0.595
6	Acknowledgement of the loss=0.903	3.06±1.11	0.729
7		3.04±1.10	0.770
8		3.20±1.10	0.712
9		2.50±1.05	0.746
10		2.84±1.05	0.700
11	Inclusion of the griever=0.796	1.59±0.90	0.429
12		2.09±1.12	0.514
13		2.03±1.06	0.530
14		1.81±0.91	0.563
15		2.30±1.23	0.432

Total Cronbach’s α value=0.888.

To determine whether there was response bias in the scale, a Hotelling’s T^2^ analysis was performed. The resulting T^2^ statistic was 1164.712, corresponding to an F-value of 80.139 and a significance level of p<0.001.

### CFA results

Prior to CFA, univariate normality was examined for each item in the CFA subsample (n=177). Skewness values ranged from −0.82 to 0.61, and kurtosis values ranged from 0.74 to 1.43, all within the acceptable thresholds of ±2 and ±7, respectively, indicating satisfactory univariate normality. Mardia’s normalised multivariate kurtosis coefficient was 3.21, falling below the threshold of 5.0, confirming the absence of meaningful multivariate non-normality. These results supported the use of ML estimation. Multivariate outlier screening using the Mahalanobis distance (D^2^) identified three cases as potential outliers (p_1_<0.001); however, removing them did not substantially alter the factor structure or fit indices, and they were retained in the final analysis to preserve sample representativeness.

The initial baseline model (three correlated factors, 15 items, 12 free factor loadings) yielded a marginal fit. Inspection of standardised factor loadings revealed that all items loaded significantly (p<0.001) on their respective factors with loadings ranging from 0.52 to 0.89, indicating no problematic items warranting removal. MI were examined, and four pairs of error covariances between conceptually related items within the same subscale were freed sequentially based on MI values exceeding 10.0 and theoretical justifiability (eg, item pairs within the ‘Acknowledgement of the Loss’ subscale sharing overlapping situational content). These modifications improved model fit without compromising the factor structure.

**Figure 1:**
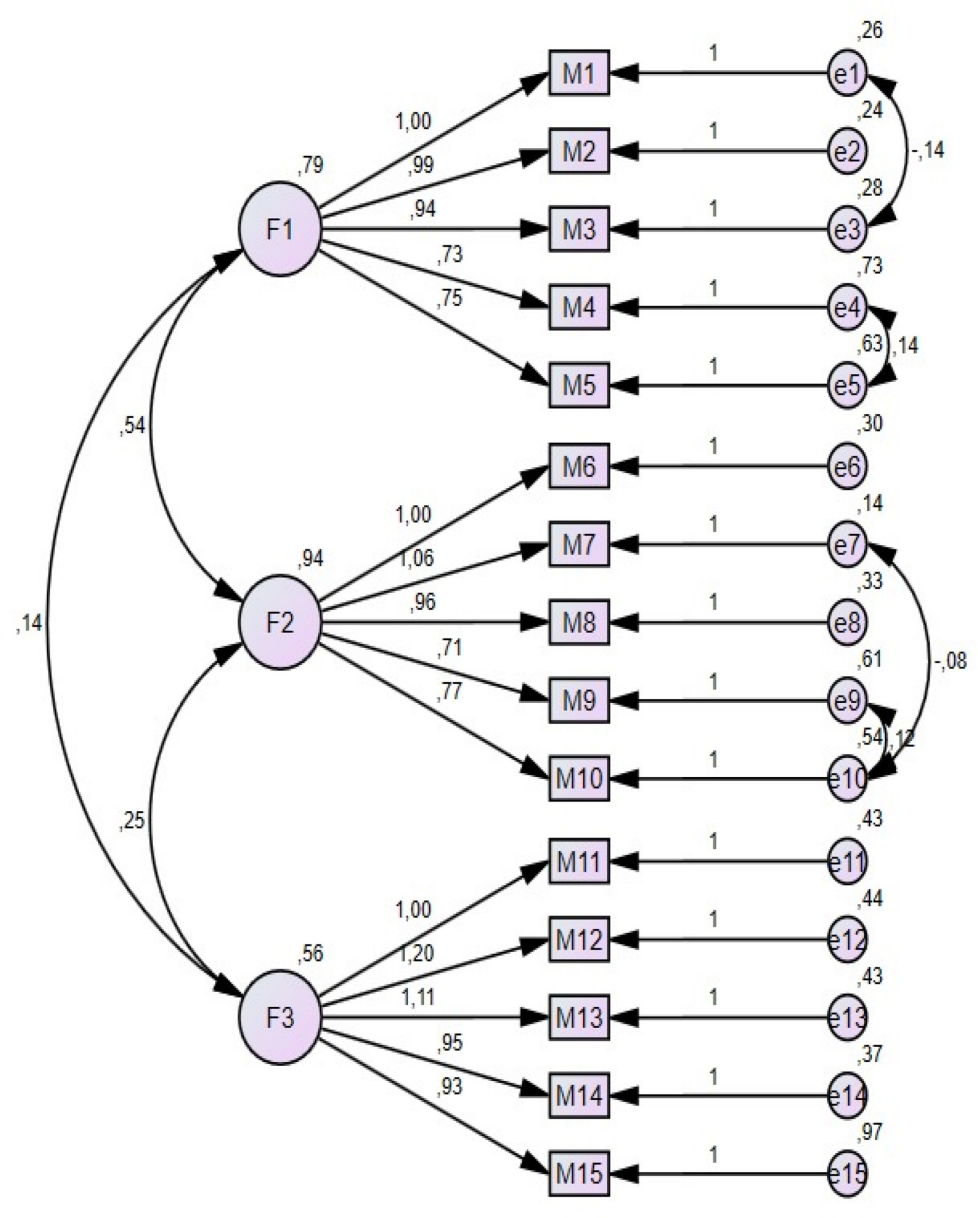
Path diagram and factor loads of the three-factor models of the scale.

The final CFA model demonstrated acceptable fit across multiple indices. The χ^2^ statistic was χ^2^(83)=338.204; although statistically significant, it is known to be sensitive to sample size and model complexity. The χ^2^/df ratio was 4.075, which, although slightly above the ideal threshold of 3.0, remains within the acceptable range for complex psychometric models. The RMSEA was 0.093, marginally exceeding the conventional 0.08 cut-off but falling within the commonly cited upper boundary of 0.10 for acceptable fit, particularly given model complexity. The remaining incremental and absolute fit indices all met (SRMR=0.062) or exceeded the 0.90 threshold: GFI=0.892, CFI=0.922, TLI=0.901, IFI=0.923 and NFI=0.900 (table 4, figure 1).

**Table 4:** Confirmatory factor analysis model fit indices for the three-factor structure of the scale (n=177)

	χ^2^	df	χ^2^/df	SRMR	RMSEA	GFI	IFI	CFI	NFI	TLI
Initial	411.761	87	4.733	0.068	0.103	0.868	0.901	0.901	0.878	0.880
Modified model	338.204	83	4.075	0.062	0.093	0.892	0.923	0.922	0.900	0.901
Acceptable threshold	–	–	≤5.00	<0.08	<0.08	≥0.90	≥0.90	≥0.90	≥0.90	≥0.90

CFI, Comparative Fit Index; df, degrees of freedom; GFI, Goodness of Fit Index; IFI, Incremental Fit Index; NFI, Normed Fit Index; RMSEA, root mean square error of approximation; SRMR, standardised root mean square residual; TLI, Tucker-Lewis Index.

### Split-half and internal consistency reliability results

To evaluate the internal reliability of the scale and its subdimensions, split-half reliability was calculated in addition to Cronbach’s α. Cronbach’s α for the total scale was 0.888. Subdimension α’s were 0.903, 0.874 and 0.796, respectively. For the first factor, Cronbach’s α was 0.894 and 0.797 for the two halves. The Spearman-Brown coefficient was 0.668, the Guttman split-half coefficient was 0.653 and the correlation between the two halves was 0.502.

### Convergent validity

Since the data demonstrated normal distribution, Pearson correlation analysis was conducted to examine the relationships between the variables. All subdimensions of the GSHCS were significantly and positively correlated with PBS-SBR, with the highest correlation observed for acknowledgement of the loss (r=0.401, p<0.05), followed by recognition of the relationship (r=0.340, p<0.05) and inclusion of the griever (r=0.176, p<0.05). The total GSHCS score was also moderately correlated with PBS-SBR (r=0.436, p<0.05). Weak but statistically significant correlations were found between recognition of the relationship and acknowledgement of the loss subdimensions and PBS-AGC (r=0.116 and r=0.054, respectively; p<0.05), while other associations with PBS-AGC were not statistically significant (table 5).

**Table 5:** Relations between GSHCS and PBS-SBR, PBS-AGC (n=355)

Variables	PBS-SBR	PBS-AGC
Recognition of the relationship	0.340*	0.116*
Acknowledgement of the loss	0.401*	0.054*
Inclusion of the griever	0.176*	0.094
Total GSHCS	0.436*	0.112

* p<0.05.

GSHCS, Grief Support in Healthcare Scale; PBS-AGC, Professional Bereavement Scale Accumulated Global Changes Subscale; PBS-SBR, Professional Bereavement Scale Short-term Bereavement Reactions Subscale.

### CR, convergent and discriminant construct validity

CR values exceeded the recommended threshold of 0.70 for all three factors: recognition of the relationship (CR=0.876), acknowledgement of the loss (CR=0.904) and inclusion of the griever (CR=0.857), confirming adequate construct reliability. AVE values also met the recommended criterion of ≥0.50 for all factors: 0.590, 0.658 and 0.548, respectively, supporting convergent validity. Discriminant validity was assessed using the Fornell-Larcker criterion. The square root of AVE for each factor (0.768, 0.811 and 0.740, respectively) exceeded all corresponding inter-factor correlations (r=0.214–0.640), providing evidence that the three factors are empirically distinct constructs.

## Discussion

The Turkish version of the GSHCS demonstrated excellent content validity, reflecting strong consensus among the expert panel regarding the clarity, relevance and representativeness of the items. Both I-CVI and S-CVI exceeded the thresholds recommended in the literature, indicating that the items are conceptually appropriate and culturally adapted to the Turkish healthcare setting.^35^ According to methodological guidelines, such high CVI scores suggest that the scale captures the intended construct with clarity and coherence, and that no fundamental conceptual gaps exist between the original instrument and its Turkish adaptation.^36 37^ This is particularly noteworthy given that grief support practices and their cultural expression can vary considerably across healthcare systems. In Turkey, where institutional support for nurses’ grief remains largely absent, and no formal regulatory framework governs nurses’ participation in patient funeral ceremonies, achieving strong content validity required careful attention to cultural nuances during translation and expert review. The fact that all items were retained without revision following expert evaluation, with only minor wording revisions for clarity and comprehensibility, further attests to the conceptual robustness of the original scale and the appropriateness of its adaptation. This strong content validity provides a solid foundation for the subsequent construct validity and reliability analyses, supporting the overall psychometric integrity of the adapted instrument.

The three-factor structure identified through EFA recognition of the relationship, acknowledgement of the loss and inclusion of the griever demonstrated strong conceptual coherence and is fully consistent with the original factor solution reported by Anderson *et al*.^11^ This structural replication across a culturally distinct sample is particularly meaningful, as it suggests that the underlying construct of grief support in healthcare transcends cultural boundaries and retains its theoretical integrity in the Turkish nursing context. Each factor captured a distinct yet interrelated dimension of grief support: relational recognition, social acknowledgement and institutional inclusion, collectively reflecting the multidimensional nature of grief support as conceptualised in the disenfranchised grief literature.^10 11^ The adequacy of the factor solution was further supported by the proportion of total variance explained, which met recommended benchmarks for psychometric instruments in the behavioural sciences.^38^,^40^ Notably, all items loaded on their theoretically expected factors with loadings exceeding the recommended threshold, indicating that the Turkish adaptation preserved the item-factor relationships of the original scale without necessitating any structural modifications.^39^,^41^ These findings collectively support the construct validity of the Turkish GSHCS and provide a sound empirical basis for its use in assessing grief support perceptions among nurses in Turkey.

The reliability analyses provided strong evidence of the psychometric soundness of the Turkish GSHCS. All subscales demonstrated acceptable to excellent internal consistency, with the ‘Acknowledgement of the Loss’ subscale exhibiting the highest item homogeneity, reflecting the conceptually focused and emotionally salient nature of this dimension in nursing practice. The ‘Recognition of the Relationship’ subscale also showed high reliability, consistent with the premise that items assessing perceived closeness between nurses and patients constitute a well-defined, internally coherent construct.^42^ The slightly lower but still acceptable reliability observed for the ‘Inclusion of the Griever’ subscale may reflect the greater heterogeneity of this dimension, which encompasses a broader range of institutional behaviours from informing staff about deaths to organising memorial services compared with the more interpersonally focused subscales. As noted by Kılıç (2016), α values in the range of 0.70–0.90 represent an optimal balance between internal consistency and item diversity, suggesting that this subscale captures meaningful variation without redundancy.^43^ Item-total correlations across all subscales surpassed the minimum threshold recommended in the literature, confirming that each item contributes meaningfully to its respective factor.^44^ Furthermore, the significant Hotelling’s T^2^ result indicates that item means differ significantly from one another, suggesting the presence of differential item endorsement patterns among participants. While this may partly reflect the scale’s multidimensional content, in which items across different subscales naturally elicit varying levels of agreement, it should also be interpreted cautiously as a potential indicator of response tendencies, which warrants attention in future applications of the instrument.^42 44 45^

CFA confirmed the three-factor structure of the Turkish GSHCS, with acceptable model fit, providing empirical support for the instrument’s structural validity. Although the χ^2^ statistic was significant and the RMSEA was marginally above the conventional threshold, these outcomes are well documented in the literature as common in large-sample psychometric studies, where the χ^2^ statistic is known to be highly sensitive to sample size and model complexity.^46 47^ The incremental fit indices, including SRMR, CFI, TLI, IFI and NFI, consistently met or exceeded recommended criteria, which are considered more robust indicators of model adequacy when evaluating latent constructs in psychometric instruments.^47^ According to best practices in CFA, a nuanced, multifaceted approach to fit evaluation is essential, as no single index provides a complete picture of model adequacy.^46^,^48^ The convergence of multiple fit indices above recommended thresholds, despite the slightly elevated RMSEA and χ^2^/df, is therefore interpreted as indicative of acceptable structural fit. Importantly, the three-factor solution, confirmed through CFA, mirrors the original factor structure reported by Anderson *et al*^11^ and the structure replicated in the Korean validation study,^24^ suggesting that the theoretical model of grief support in healthcare generalises across culturally distinct nursing contexts. This cross-cultural structural consistency strengthens confidence in the construct validity of the Turkish GSHCS and supports its use as a theoretically grounded measurement instrument in grief support research.

Both Cronbach’s α and split-half reliability analyses consistently supported the internal consistency of the Turkish GSHCS across the total scale and all subscales, confirming that items within each dimension measure the same underlying construct.^49 50^ The slightly lower reliability observed for the ‘Inclusion of the Griever’ subscale mirrors the pattern reported in the original scale development study by Anderson *et al*^11^ and is more likely attributable to the broader, institutionally oriented nature of this dimension than to a measurement weakness. These findings indicate that the Turkish GSHCS demonstrates sufficient internal consistency and measurement stability for use in clinical research and organisational assessment contexts.^44^

Convergent validity was supported through significant positive associations between all GSHCS subscales and the PBS-SBR, confirming theoretically meaningful alignment between the two instruments. This pattern is conceptually coherent: PBS-SBR captures acute grief reactions experienced by healthcare professionals following patient loss, which are directly related to the support-focused dimensions assessed by the GSHCS. Among the subscales, ‘Acknowledgement of the Loss’ demonstrated the strongest association with PBS-SBR, a finding theoretically interpretable, given that explicit recognition of grief by colleagues and supervisors is likely to be most salient during the acute phase of bereavement.^11^ Convergent validity was supported through significant positive associations between all GSHCS subscales and the PBS-SBR, confirming theoretically meaningful alignment between the two instruments. Among the subscales, ‘Acknowledgement of the Loss’ demonstrated the strongest association with PBS-SBR, a finding theoretically interpretable, given that explicit recognition of grief by colleagues and supervisors is likely to be most salient during the acute phase of bereavement.^11^ Correlations with PBS-AGC, however, were negligibly low and are insufficient to support convergent validity with this subscale. PBS-AGC assesses long-term cumulative changes in professional identity and worldview following repeated patient losses, a construct that is theoretically more distal from the immediate support-focused content of the GSHCS.^30 31^ The absence of meaningful convergence with PBS-AGC therefore reflects a boundary condition of the scale’s nomological network rather than a validity failure. Future studies should examine convergent validity using instruments more directly aligned with workplace grief support, such as measures of social support, compassion fatigue or burnout, to more comprehensively map the construct’s nomological network.^49 50^

### Implications for practice

The validated Turkish version of the GSHCS provides a much-needed tool for assessing how grief support is perceived within healthcare environments. From a clinical perspective, this instrument can help managers and organisational leaders identify gaps in institutional grief practices, enabling the design of targeted support programmes such as peer debriefing, memorial services or grief counselling. The three-factor structure, Recognition of the Relationship, Acknowledgement of the Loss and Inclusion of the Griever, offers insight into not only interpersonal and emotional aspects of grief but also organisational systems and culture. Regularly using the scale in staff assessments could foster a more compassionate workplace, reduce moral distress and support psychological well-being among nurses exposed to patient loss. In continuing professional development, the scale may serve as a pre-intervention and post-intervention measure to evaluate the effectiveness of grief literacy or resilience training programmes.

A particularly noteworthy finding from the sample characteristics is that only 20% of participants reported having access to workplace institutional grief support services. This figure highlights a critical gap in organisational support structures within Turkish healthcare settings and has direct implications for the interpretation of GSHCS scores. Nurses practising in environments devoid of formal grief support mechanisms are likely to score lower on the 'Inclusion of the Griever’ subscale, which assesses institutionally organised grief practices such as memorial services and death notifications. This may restrict score variability and affect the instrument’s sensitivity in contexts where grief support is largely absent. Beyond its psychometric implications, this finding underscores the urgent need for policy-level interventions to establish structured grief support programmes in Turkish healthcare institutions and reinforces the practical utility of the GSHCS as a diagnostic tool for identifying and addressing these institutional deficiencies.

### Limitation

This study has some limitations. First, the sample was recruited via convenience sampling through online platforms, which may limit the representativeness of the findings across all nursing specialties and geographic regions of Turkey. Future studies should consider probability-based sampling strategies to enhance the generalisability of the psychometric findings. Second, test-retest reliability was assessed only in the pilot subsample (n=30), limiting conclusions about the long-term temporal stability of the Turkish GSHCS; future validation studies with larger samples and extended retest intervals are warranted. Finally, convergent validity was assessed using a single external instrument, limiting triangulation of the construct. Future studies should incorporate additional theoretically related measures, such as instruments assessing workplace social support, burnout or compassion fatigue to more comprehensively map the nomological network of the GSHCS within the Turkish nursing context.

## Conclusion

This study established the Turkish version of the GSHCS as a valid and reliable instrument for assessing perceptions of grief support among nurses. The scale demonstrated strong content validity, acceptable factor structure and high internal consistency through rigorous translation, cultural adaptation and psychometric evaluation. The findings confirm that the Turkish version of the GSHCS is a psychometrically sound instrument for assessing nurses’ perceptions of grief support in healthcare settings. Its potential utility in clinical, educational and research contexts warrants further investigation in future studies.

The findings confirm that the Turkish version of the GSHCS is a psychometrically sound instrument for assessing nurses’ perceptions of grief support in healthcare settings. Its potential utility in clinical, educational and research contexts warrants further investigation. Future studies should extend validation to other healthcare professional groups, test measurement invariance across clinical subgroups and employ the scale as an outcome measure in grief support interventions. National multicentre studies with probability-based sampling would further strengthen the generalisability of these findings and contribute to the development of evidence-based institutional grief support policies in Turkey.

## Data Availability

Data are available upon reasonable request.
