# Supplementary material for: Adaptation and psychometric validation of the Turkish version of the Grief Support in Healthcare Scale: a methodological study
Source: BMJ Open. 2026 Jul 3;16(7):e116014. doi: 10.1136/bmjopen-2025-116014 (PMC13343047; doi:10.1136/bmjopen-2025-116014)
Supplement: Supplementary file 1 [file bmjopen-2025-116014.reviewer_comments.pdf]

PEER REVIEW HISTORY

BMJ Open publishes all reviews undertaken for accepted manuscripts. Reviewers are asked to complete a checklist review form and are provided with free text boxes to elaborate on their assessment. These free text comments are reproduced below.

ARTICLE DETAILS

Title (Provisional)

Adaptation and Psychometric Validation of the Turkish Version of the Grief Support in Healthcare Scale: A Methodological Study from Türkiye

Authors

Şimşek Arslan, Buket; Semerci Şahin, Remziye

VERSION 1 - REVIEW

**Reviewer**	**1**
**Name**	**Ay, Ayşe**
**Affiliation**	**Başkent Üniversitesi, Pediatric Nursing**
**Date**	**02-Feb-2026**
**COI**	**None**

I believe that this study makes a meaningful contribution to the existing literature by providing a culturally adapted and psychometrically validated instrument to assess grief support in healthcare settings within the Turkish context. Grief support is a critical yet often underexplored component of healthcare, particularly for nurses who are frequently exposed to patient loss and bereavement.

By adapting the Grief Support in Healthcare Scale into Turkish and rigorously evaluating its validity and reliability, this study addresses an important methodological gap and offers a robust tool for both clinical research and organizational assessment. The findings may support future research, inform institutional policies, and contribute to improving supportive environments for healthcare professionals.

Given the increasing recognition of the emotional burden experienced by nurses, i believe that the topic is timely, relevant, and of significant importance to the field of healthcare research and practice

---

**Reviewer**	**2**
**Name**	**Tagharrobi, Zahra**

Affiliation **Kashan University of Medical Sciences, Trauma Nursing Research Centre, Faculty of Nursing and Midwifery**

Date **05-Feb-2026**

COI **None**

I hope this message finds you well.
The manuscript has been reviewed, and the following issues should be addressed in the revised version:

- In the *Results* section of the abstract, interpretive statements should be avoided. Model fit indices from the confirmatory factor analysis (CFA) should be reported.
- In the introduction, the background of the tool should be clearly described, including the context and process of its development, its strengths in comparison with similar tools, the availability of versions in other languages, and its application in other countries.
- The sample size and characteristics should be reported separately for the exploratory factor analysis (EFA) and confirmatory factor analysis (CFA).
- The sampling method should be explicitly stated in both the abstract and the main text.
- The validity and reliability of the nurse information form should be reported.
- The primary purpose of the pilot study is to identify inappropriate items through item analysis; assessing reliability alone is not appropriate at this stage. tool validity should first be examined and confirmed, followed by reliability assessment. If the aim was to evaluate the stability of the tool, this should be assessed and reported at the end stage of validity and internal consistency evaluation using a small subsample.
- It should be noted that principal component analysis (PCA) and exploratory factor analysis are distinct methodologies; therefore, careful consideration should be given to the choice of extraction method in EFA.
- Sample characteristics for EFA and CFA should be presented separately. Although the text indicates that EFA was conducted on half of the sample and CFA on the other half, this distinction is not clearly reflected in the content or titles of the tables.
- In the Results section, interpretation and subjective judgment should be avoided.
- In Table 4, the acceptable thresholds for model fit indices, as well as their values before and after model modification, should be reported.
- Table 5 is unnecessary, and its content should be incorporated into the text.
- In Table 6, the types of statistical tests used should be clearly specified.
- The CFA diagram has been presented twice and should be corrected.
- As this is a psychometric tool study, the reporting should adhere to the COSMIN checklist.
- Missing data and the methods used to handle them in both types of factor analysis should be clearly reported.
- Detailed information regarding the CFA procedure should be provided, including the parameter estimation method, assessment of univariate and multivariate normality, evaluation of outliers, identification of problematic items, and the methods employed for model modification.
- Composite reliability, as well as convergent and discriminant construct validity, should be reported.

- Discussion of prerequisites for exploratory factor analysis is unnecessary in the Discussion section.
- Repetition of results and methodological details should be avoided in the Discussion.
- The Discussion section is weak and should place greater emphasis on the instrument's structure and its conceptual interpretation.
- The stated limitation concerning the cross-sectional design, causal relationships among variables, and the recommendation for longitudinal studies is not relevant to the present study.
- Appropriate and relevant recommendations for future research should be provided.
- Some references are outdated, and the total number of references is excessive.

**Reviewer**	**3**
**Name**	**Yıldız, Hatice**
**Affiliation**	**Aydin Adnan Menderes Universitesi, Pediatirc Nurses**
**Date**	**23-Apr-2026**
**COI**	**None**

The topic is highly meaningful and interesting, and the study offers potentially important insights into the cultural adaptation and psychometric validation of the Grief Support in Healthcare Scale for Turkish nurses. However, several methodological concerns raise questions about the validity and interpretation of the results.

Abstract

Change the phrase "This methodological study." to "This study is a methodological study" or simply "Methodological study."

Keywords

Not MeSH compliant.

Introduction

It is recommended not to start directly with a heading. Begin with a short sentence stating the importance of the topic, then continue with subheadings.

Method

Studydesign

As noted in the discussion, this study is also cross sectional. The study design should be written as "methodological, cross sectional study."

Studysetting

The phrase "online platforms" should be clarified. Specify which platforms were used. Was convenience sampling employed?

Inclusioncriteria

Remove "volunteer to participate in the study." Since the scale used includes recognition of grief experience after patient loss, it would be logical to include nurses who have experienced at least one patient death. Either clarify this criterion or provide a justification for why it was not included.

Data collection tools

• Nurse Information Form: appears twice consecutively. Provide a reference for how the items were decided.

• PBS: Permission from the authors of the Turkish adaptation or the original scale developers should be stated. Reference 30 is given, but permission information is missing.

Datacollection

Clearly detail the method of reaching participants.

Content validity

• "were made to the draft following expert recommendations" – what specific changes were made?

• The authors note that the "Inclusion of the Griever" subscale was removed in the Korean version, but all items were retained in this study because there is no specific regulation in Turkey. It is recommended to rephrase this paragraph as:

"Since there is no legal regulation regarding nurses' participation in patient funeral ceremonies in Turkey, all items were included in the analysis to preserve the integrity of the scale. However, the cultural appropriateness of this subscale should be re evaluated in future studies."

• It is recommended to add the SRMR value to the model fit indices. In Figure 1 (page 34), AGFI = 0.844 is given, but this value is not present in Table 4.

Results

• Test retest was performed on 30 nurses, but the test retest correlation coefficient (ICC or Pearson's r) is not reported.

• Table 3: Under the "Item Total Correlation" column, values are only given for the Recognition subscale. Values should also be provided separately for Acknowledgement and Inclusion. Currently, it appears misleading.

• The same diagram appears on pages 33 and 34. One should be removed.

Discussion

• The interpretation of Hotelling's T^2 test should be checked. The test is significant. A significant Hotelling's T^2 indicates that the mean differences among items are different from zero, which may suggest response bias.

• Correlations with PBS AGC are very low (r = 0.054, 0.116) and some are practically meaningless. The authors state that "statistical significance indicates partial conceptual overlap." However, such low correlations ($r < 0.10$) are not sufficient for convergent validity. The meaning of the low PBS AGC correlations in terms of convergent validity should be discussed more realistically.

• Table 1. "Having grief support service in the hospital" – only 20% of participants reported having such a service. The impact of this should be discussed.

Limitation

"Longitudinal research is needed to explore how grief support perceptions evolve and influence emotional well-being or job performance." This statement is not a valid limitation for a methodological (psychometric) study. The main purpose of this study is to test the validity and reliability of the Turkish version of the Grief Support in Healthcare Scale. It should be removed.

VERSION 1 - AUTHOR RESPONSE

**Reviewer#2 Comment**	**Author Response**
In the Results section of the abstract, interpretive statements should be avoided. Model fit indices from the confirmatory factor analysis (CFA) should be reported.	We thank the reviewer for this constructive comment. In response, we have revised the Results section of the abstract by removing interpretive statements and replacing them with objective, quantitative reporting. Specifically, the CFA model fit indices are now explicitly reported ($\chi^2(83) = 338.204$, $\chi^2/df = 4.075$, RMSEA = 0.093, GFI = 0.892, CFI = 0.922, TLI = 0.901, IFI = 0.923, NFI = 0.900), and descriptive language such as "excellent," "high," and "acceptable" has been removed throughout the Results section of the abstract.
In the introduction, the tool's background should be clearly described, including its development context and process, its strengths relative to similar tools, the availability of versions in other languages, and its application in other countries.	We thank the reviewer for this important suggestion. In the revised manuscript, we have added a dedicated paragraph to the Introduction (under the "Need for a Measurement Tool in Türkiye and Aim of the Study" subsection) that comprehensively describes the GSHCS's development context and process (Anderson et al., 2010; long-term care settings, disenfranchised grief framework), its distinguishing strength as a support-focused rather than reaction-focused instrument, and its cross-cultural adaptation in South Korea (Ahn et al., 2023), including the culturally driven structural modification applied in that validation. This background now precedes the

	study aim, providing readers with a clear rationale for selecting this instrument for Turkish adaptation. *(See revised Introduction, paragraph 3 of the "Need for a Measurement Tool" section.)*
The sample size and characteristics should be reported separately for the exploratory factor analysis (EFA) and confirmatory factor analysis (CFA).	We thank the reviewer for this important methodological comment. In response, we have clarified the sample sizes for each analysis throughout the manuscript. The 355 participants were randomly split into two equal halves: EFA was conducted on the first half (n = 178) and CFA on the second half (n = 177). This information has been added to the Data Analysis section of the Methods, the Participants section of the abstract, and the captions for Tables 2 (EFA) and 4 (CFA).
The sampling method should be explicitly stated in both the abstract and the main text.	We thank the reviewer for this suggestion. The sampling method has been explicitly identified as convenience sampling in both the abstract (Participants section) and the main text (Study Setting section).
The validity and reliability of the nurse information form should be reported.	We respectfully clarify that the Nurse Information Form is a researcher-developed sociodemographic questionnaire comprising 7 descriptive items (e.g., age, gender, educational level, years of experience, clinical setting, grief education status, and availability of workplace grief support). Such forms are not psychometric instruments and therefore are not subject to validity and reliability analyses, as they do not measure a latent construct. This is consistent with standard methodological practice in scale adaptation and survey-based nursing research, where sociodemographic data collection forms are distinguished from outcome measurement tools.[25–28] Accordingly, no psychometric evaluation of the Nurse Information Form was performed or reported. The form is described in the Data Collection Tools section solely to document the variables collected for sample characterization purposes.
The primary purpose of the pilot study is to identify inappropriate items through item analysis; assessing reliability alone is not appropriate at this stage. tool's validity should first be examined and confirmed, followed by a reliability assessment. If the aim was to evaluate the stability of the tool, it should be assessed and reported at the end of the validity and internal consistency evaluation using a small subsample.	We thank the reviewer for this important methodological observation. The pilot study section has been substantially revised to reflect recommended psychometric practice. The revised text clarifies that the primary purpose of the pilot study was item analysis: 30 nurses completed the scale and provided feedback on the readability, comprehensibility, and appropriateness of each item, which guided final

	revisions before the main study. The test–retest assessment (day 0 and day 14) is now described as a secondary aim conducted on the pilot subsample at the end of the pilot phase, following content validity confirmation and item refinement, to evaluate the instrument's temporal stability. This sequential structure is now explicitly stated in the manuscript.
It should be noted that principal component analysis (PCA) and exploratory factor analysis are distinct methodologies; therefore, careful consideration should be given to the choice of extraction method in EFA.	We thank the reviewer for this important methodological point. The reviewer is correct that principal component analysis (PCA) and exploratory factor analysis (EFA) are conceptually distinct techniques. PCA is a data reduction method that does not model latent structure, whereas EFA specifically aims to identify underlying common factors. In response, we reran the analysis using Principal Axis Factoring (PAF) as the extraction method, an appropriate and widely used approach within the EFA framework. The three-factor structure was fully retained, confirming the robustness of the original findings. All references to "principal component analysis" have been replaced with "Principal Axis Factoring (PAF)" throughout the manuscript. The total explained variance has been updated accordingly (58.33%).
Sample characteristics for EFA and CFA should be presented separately. Although the text indicates that EFA was conducted on half of the sample and CFA on the other half, this distinction is not clearly reflected in the table content or titles.	We thank the reviewer for this comment. We agree that the distinction between the EFA and CFA subsamples should be clearly reflected in the manuscript. Accordingly, the titles of Tables 2 and 4 have been updated to specify the respective sample sizes (Table 2: n = 178 for EFA; Table 4: n = 177 for CFA). In addition, the Data Analysis section explicitly states that the 355 participants were randomly split into two equal halves: EFA was performed on the first half (n = 178) and CFA on the second half (n = 177). These changes ensure that the distinction between the subsamples is transparent throughout the manuscript.
In the Results section, interpretation and subjective judgment should be avoided.	We thank the reviewer for this important comment. In response, we have carefully revised the Results section to remove all interpretive and evaluative language. Specifically, subjective descriptors such as "excellent," "high," "acceptable," "marginal," "meritorious," and "satisfactory" have been removed. Phrases such as "indicating strong item–factor associations," "supporting the factorability of the correlation matrix," "demonstrating a high level of consistency," and

	"further supporting the adequacy of the model" have also been deleted. All findings are now reported as objective numerical values without accompanying qualitative judgments.
In Table 4, the acceptable thresholds for model fit indices, as well as their values before and after model modification, should be reported.	Acceptable thresholds for model fit indices, as well as their values before and after model modification values were added in Table 4.
• Table 5 is unnecessary, and its content should be incorporated into the text.	Thank you for this valuable suggestion. In line with your recommendation, Table 5 has been removed, and the relevant findings have been integrated into the Results section of the manuscript.
In Table 6, the types of statistical tests used should be clearly specified.	Thank you for this valuable comment. The type of statistical test used in Table 6 has now been clearly specified. Since the data showed normal distribution, Pearson correlation analysis was used, and this information has been added to the table note.
The CFA diagram has been presented twice and should be corrected.	It was corrected.
As this is a psychometric tool study, the reporting should adhere to the COSMIN checklist.	We thank the reviewer for this important methodological recommendation. We agree that psychometric studies should adhere to COSMIN (COnsensus-based Standards for the selection of health Measurement INstruments) reporting standards. Upon careful review, we have revised the manuscript to align with COSMIN guidelines.
Missing data and the methods used to handle them in both types of factor analysis should be clearly reported.	Thank you for this important comment. Information on handling missing data has been added to the Data Analysis section. No missing data were identified in the dataset prior to conducting EFA and CFA analyses.
Detailed information regarding the CFA procedure should be provided, including the parameter estimation method, assessment of univariate and multivariate normality, evaluation of outliers, identification of problematic items, and the methods employed for model modification.	We thank the reviewer for this constructive comment. In response, we have substantially expanded CFA reporting across the Methods (Data Analysis) and Results sections. The revised manuscript now includes the following procedural details: (1) univariate normality was assessed via item-level skewness and kurtosis values, and multivariate normality was evaluated using Mardia's normalized multivariate kurtosis coefficient, both confirming the suitability of Maximum Likelihood (ML) estimation; (2) multivariate outliers were identified using Mahalanobis distance (D^2), and their influence on model fit was examined prior to the final analysis; (3)

	standardized factor loadings were inspected for all items, and no items were identified as problematic (all $\lambda \geq .50$, $p < .001$); and (4) model modification was guided by modification indices (MI > 10.0), with error covariance terms freed only when theoretically justifiable. These additions have been incorporated into the manuscript (see revised Methods, Data Analysis section, and Results, Confirmatory Factor Analysis section).
Composite reliability, as well as convergent and discriminant construct validity, should be reported.	We thank the reviewer for this important methodological recommendation. In the revised manuscript, composite reliability (CR) and average variance extracted (AVE) have been calculated for all three factors and reported in a dedicated section following the CFA results. CR values ranged from .857 to .904, and AVE values ranged from .548 to .658, all meeting the recommended thresholds of ≥ .70 and ≥ .50, respectively, confirming adequate construct reliability and convergent validity. Discriminant validity was assessed using the Fornell-Larcker criterion; the square root of AVE for each factor exceeded all corresponding inter-factor correlations, supporting the empirical distinctiveness of the three factors. A summary table presenting CR, AVE, √AVE, and inter-factor correlations has been added to the manuscript
Discussion of prerequisites for exploratory factor analysis is unnecessary in the Discussion section.	We thank the reviewer for this comment. The discussion of EFA prerequisites (KMO, Bartlett's Test, eigenvalue criteria, and rotation justification) has been removed from the Discussion section. The revised paragraph now focuses exclusively on the interpretive and conceptual implications of the three-factor structure in relation to the original scale and the broader literature.
Repetition of results and methodological details should be avoided in the Discussion.	We thank the reviewer for this valuable feedback. The Discussion section has been carefully revised to eliminate repetition of numerical results and methodological details already presented in the Results section. Specific values for fit indices, factor loadings, eigenvalues, Cronbach's alpha, and correlation coefficients have been removed from the Discussion. Each paragraph now focuses solely on the interpretation and contextualization of findings in relation to the existing literature and the original scale.

The Discussion section is weak and should place greater emphasis on the instrument’s structure and its conceptual interpretation.	We thank the reviewer for this feedback. The Discussion section has been substantially revised to move beyond numerical reporting and place greater emphasis on the conceptual interpretation of the instrument's three-factor structure. Each paragraph now addresses the theoretical significance of the findings, contextualises the subscale-level results within the disenfranchised grief and nursing bereavement literature, and draws comparisons with the original scale and existing cross-cultural validation studies. These revisions collectively strengthen the conceptual depth and interpretive quality of the Discussion.
The stated limitation concerning the cross-sectional design, causal relationships among variables, and the recommendation for longitudinal studies is not relevant to the present study.	We thank the reviewer for this valid observation. We agree that the limitations regarding the cross-sectional design and the inability to draw causal inferences are not applicable to a methodological scale adaptation study, which does not aim to examine causal relationships among variables. This statement has been removed from the Limitations section in the revised manuscript. The remaining limitations now accurately reflect the scope of the present study, focusing on sample representativeness, the size of the test–retest subsample, and the use of a single external instrument for convergent validity assessment.
Appropriate and relevant recommendations for future research should be provided.	We thank the reviewer for this constructive suggestion. In the revised manuscript, we have added a dedicated future research paragraph at the end of the conclusion.
Some references are outdated, and the total number of references is excessive	All references were carefully reviewed and updated in accordance with the journal's submission guidelines. As the guidelines specify a maximum number of allowable references, the reference list has been revised to comply with this requirement while ensuring that all cited sources remain relevant and essential to the manuscript.
**Reviewer#3 Comment**	**Author Response**
Abstract<br>Change the phrase "This methodological study." to "This study is a methodological study" or simply "Methodological study."	Thank you so much for your contribution. The abstract was revised based on the your comments.
Keywords<br>Not MeSH compliant.	We thank the reviewer for this observation. The keywords have been revised to ensure full MeSH (Medical Subject Headings) compliance

Introduction<br>It is recommended not to start directly with a heading. Begin with a short sentence stating the importance of the topic, then continue with subheadings.	We thank the reviewer for this suggestion. In the revised manuscript, the Introduction now opens with two brief sentences establishing the clinical and research significance of the topic before proceeding to the subheadings. This revision improves the overall flow and contextualizes the subsequent sections for the reader.
Method<br>Studydesign<br>As noted in the discussion, this study is also cross-sectional. The study design should be written as "methodological, cross-sectional study."	Thank you so much for your suggestion. The study design was revised based on the your comments.
Studysetting<br>The phrase "online platforms" should be clarified. Specify which platforms were used. Was convenience sampling employed?	We thank the reviewer for this observation. The Study Setting section has been revised accordingly. The vague reference to "online platforms" has been replaced with a specific list of recruitment channels: WhatsApp groups, LinkedIn, Facebook groups, Instagram, and the e-mail lists of nursing professional associations. The use of convenience sampling has also been explicitly stated in the revised text.
Inclusioncriteria<br>Remove "volunteer to participate in the study." Since the scale used includes recognition of grief experience after patient loss, it would be logical to include nurses who have experienced at least one patient death. Either clarify this criterion or provide a justification for why it was not included.	We thank the reviewer for this methodologically important observation. The phrase "volunteer to participate in the study" has been removed from the inclusion criteria. Furthermore, we confirm that prior patient death experience was indeed assessed in the Nurse Information Form; participants were required to have experienced at least one patient death during their career. This criterion has now been explicitly added to the inclusion criteria in the revised manuscript, strengthening the conceptual alignment between the sample characteristics and the construct measured by the GSHCS.
Data collection tools<br>• Nurse Information Form: appears twice consecutively. Provide a reference for how the items were decided.<br>• PBS: Permission from the authors of the Turkish adaptation or the original scale developers should be stated. Reference 30 is given, but permission information is missing.	We thank the reviewer for these observations. First, the consecutive repetition of "Nurse Information Form" has been corrected in the revised manuscript. Additionally, a reference has been added to indicate that the items of the Nurse Information Form were determined based on a review of the relevant literature on grief in nursing and workplace bereavement support. Second, regarding the PBS, we confirm that permission to use the Turkish adaptation of the scale was obtained from Eryüksel and Özbaş[30] prior to data collection. This information has now been explicitly stated in the Data Collection Tools section of the revised manuscript.

Data collection<br>Clearly detail the method of reaching participants.	We thank the reviewer for this observation. The Data Collection section has been substantially revised to provide a transparent and detailed account of the participant recruitment process. The revised text specifies that prior permission was obtained from group and platform administrators before distribution, that the survey was shared exclusively through professional nursing groups on WhatsApp and Facebook, as well as via LinkedIn and Instagram, and that nursing professional associations facilitated dissemination through their own WhatsApp networks. The informed consent procedure, data integrity measures, and the 100% completion rate are also described in the revised section.
Content validity<br>• "were made to the draft following expert recommendations" – what specific changes were made?<br>• The authors note that the "Inclusion of the Griever" subscale was removed in the Korean version, but all items were retained in this study because there is no specific regulation in Turkey. It is recommended to rephrase this paragraph as:<br>"Since there is no legal regulation regarding nurses' participation in patient funeral ceremonies in Turkey, all items were included in the analysis to preserve the integrity of the scale. However, the cultural appropriateness of this subscale should be re-evaluated in future studies."<br>• It is recommended to add the SRMR value to the model fit indices. In Figure 1 (page 34), AGFI = 0.844 is given, but this value is not present in Table 4.	We thank the reviewer for this clarifying question. Following expert recommendations, minor linguistic and cultural revisions were made to several items to improve clarity and cultural appropriateness. Specifically, certain English expressions that lacked equivalent connotations in Turkish were rephrased to better reflect the cultural context of nursing practice in Türkiye. For example, the term "residents" was contextualized to align with the Turkish healthcare setting, where nurses work across diverse inpatient environments rather than exclusively in long-term care facilities. Additionally, some sentence structures were simplified for readability without altering the conceptual meaning of the items. No items were added or removed during this process. These revisions have been clarified in the revised manuscript (see the Methods section, Content Validity).<br>Figure 1 and Table 4 were edited based on your suggestion<br><br>The SRMR result was added to Table 4.
Results<br>• Test retest was performed on 30 nurses, but the test retest correlation coefficient (ICC or Pearson's r) is not reported.<br>• Table 3: Under the "Item Total Correlation" column, values are only given for the Recognition subscale. Values should also be provided separately for Acknowledgement and Inclusion. Currently, it appears misleading.<br>• The same diagram appears on pages 33 and 34. One should be removed.	Thank you for this valuable suggestion. In response, we have added a dedicated "Test–retest reliability" subsection to the Results section, reporting both the Pearson correlation coefficient ($r = 0.857$, $p < 0.001$) and the intraclass correlation coefficient (ICC = 0.937), which confirm the excellent temporal stability of the scale. The revised text is included in the manuscript<br><br>Thank you for your careful observation. Item-total correlation values were calculated and

	presented separately for all three subscales. However, we recognize that the table layout may have caused ambiguity. Therefore, Table 3 has been revised to improve clarity and to present the subscale structure and Cronbach's alpha values more clearly.<br><br>The diagram duplication was removed.
Discussion<br>• The interpretation of Hotelling's T^2 test should be checked. The test is significant. A significant Hotelling's T^2 indicates that the mean differences among items are different from zero, which may suggest response bias.<br>• Correlations with PBS AGC are very low ($r = 0.054, 0.116$) and some are practically meaningless. The authors state that "statistical significance indicates partial conceptual overlap." However, such low correlations ($r < 0.10$) are not sufficient for convergent validity. The meaning of the low PBS AGC correlations in terms of convergent validity should be discussed more realistically.<br>• Table 1. "Having grief support service in the hospital" – only 20% of participants reported having such a service. The impact of this should be discussed.	We thank the reviewer for this critical observation. We agree that the correlations with PBS-AGC are too low to support convergent validity and that the original interpretation was overstated. The Discussion has been revised accordingly. The revised text now explicitly acknowledges that the negligibly low correlations with PBS-AGC are insufficient for convergent validity, and offers a conceptual explanation based on the theoretical distance between the two constructs. The need for future studies using more proximal instruments has also been noted.
Limitation<br>"Longitudinal research is needed to explore how grief support perceptions evolve and influence emotional well-being or job performance." This statement is not a valid limitation for a methodological (psychometric) study. The main purpose of this study is to test the validity and reliability of the Turkish version of the Grief Support in Healthcare Scale. It should be removed.	We thank the reviewer for this valid observation. We agree that this statement is not appropriate for a psychometric validation study. The sentence has been removed from the Limitations section in the revised manuscript.

VERSION 2 - REVIEW

**Reviewer**	**3**
**Name**	**Yıldız, Hatice**
**Affiliation**	**Aydin Adnan Menderes Universitesi, Pediatirc Nurses**
**Date**	**09-Jun-2026**
**COI**	

The authors have made all the required corrections. The paper is now suitable for publication. No further changes are needed.
